# *In-situ* Raman spectroscopy of amorphous calcium phosphate to crystalline hydroxyapatite transformation

**DOI:** 10.1016/j.mex.2018.09.015

**Published:** 2018-10-06

**Authors:** Jessica A. Stammeier, Bettina Purgstaller, Dorothee Hippler, Vasileios Mavromatis, Martin Dietzel

**Affiliations:** a Institute of Applied Geosciences, Graz University of Technology, Rechbauerstraße 12, 8010 Graz, Austria; b Géosciences Environnement Toulouse (GET), CNRS, UMR 5563, Observatoire Midi-Pyrénées, 14 Av. E. Belin, 31400 Toulouse, France

**Keywords:** *In-situ* Raman monitoring, Amorphous calcium phosphate, *In-situ* monitoring, Raman spectroscopy, Intermediate phase, Apatite

## Abstract

Amorphous calcium phosphate (Ca_3_(PO_4_)_2_xnH_2_O; n = 3–4.5; ACP) is a precursor phase of the mineral hydroxyapatite (Ca_5_(PO_4_)_3_(OH); HAP) that in natural settings occurs during both authigenic and biogenic mineral formation. In aqueous solutions ACP transforms rapidly to the crystalline phase. The transformation rate is highly dependent on the prevailing physico-chemical conditions, most likely on: Ca & PO_4_ concentration, pH and temperature. In this study, we conducted a calcium phosphate precipitation experiment at 20 °C and pH 9.2, in order to study the temporal evolution of the phosphate mineralogy. We monitored and assessed the transformation process of ACP to crystalline HAP using highly time-resolved *in-situ* Raman spectroscopy at 100 spectra per hour, in combination with solution chemistry and XRD data. Transformation of ACP to crystalline HAP occurred within 18 h, as it is illustrated in a clear peak shift in Raman spectra from 950 cm^−1^ to 960 cm^−1^ as well as in a sharpening of the 960 cm^−1^ peak. The advantages of this method are:

• In-situ Raman spectroscopy facilitates quasi – continuous monitoring of phase transitions.
• It is an easy to handle and non-invasive method.

## Graphical abstract

**Figure:**
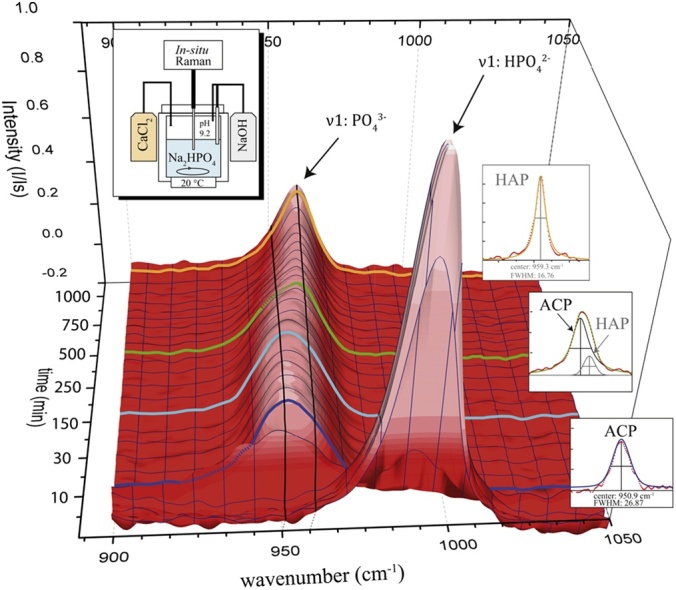

Specifications table

**Table:** 

**Subject Area**	*Earth and Planetary Sciences*
**More specific subject area:**	*Hydrogeochemistry*
**Method name:**	*In-situ* Raman monitoring

## Introduction

Hydroxyapatite (Ca_5_(PO_4_)_3_(OH); HAP, Table 1) has a wide range of occurrences and uses. Those span from products of biomineralization and post-sedimentation in natural surroundings [[1], [2], [3]], add-ons in composites for tailored properties e.g., of cements and water treatment agents [[4], [5], [6]] to innovative medical-related products e.g., for remineralization of teeth or for bone grafts [[7], [8], [9], [10]]. The formation of HAP in these settings however, may occur via the transformation of an amorphous precursor. Indeed, amorphous calcium phosphate Ca_3_(PO_4_)_2_xnH_2_O; n = 3–4.5; ACP) can often act as a precursor phase of HAP, especially during its authigenic or biogenicformation. ACP has already been identified as a transient phase during bone mineralization in the 1960ies by mere optical measures [11]. This observation was later confirmed in several studies by X-ray diffraction (e.g. [12]:). Furthermore, ACP was found during the mineralization of fish bone [13], during microbially-mediated formation of phosphatic sediments [14], or mineralization of teeth [15] highlighting the relevance of investigating ACP transformation pathways in medical, geological, biological or paleontological fields of science. In aqueous environments, the meta-stable ACP precipitates as intermediate or transitional phase, which then rapidly converts into the crystalline apatite-phase [16]. The transformation rate depends highly on the physico-chemical conditions of the solution, e.g., element availability [[17], [18], [19]], pH and temperature [[20], [21], [22]]. In alkaline media, however, transformation pathways were found to be more complex, because ACP rapidly hydrolyses to the meta-stable octacalciumphosphate (Ca_8_(PO_4_)_6_x5H_2_O; OCP), which subsequently transforms to HAP [23].

**Table 1:** Selected calcium phosphates and their respective chemical formula, molar Ca/P and *ν*1 P—O stretching in Raman spectroscopy. After Dorozhkin [32], Combes & Rey [7]; and Crane et al. [34].

Mineral	Chemical formula	Ca/P	ν1 P-O stretching
Amorphous Calcium Phosphate (ACP)	Ca_3_(PO_4_)_2_xnH_2_O, n = 3 – 4.5	1.2 – 2.5	950 cm^−1^
Octacalciumphosphate (OCP)	Ca_8_H_2_(PO_4_)_6_x5H_2_O	1.33	955 cm^−1^
Hydroxyapatite (HAP)	Ca_5_(OH)(PO_4_)_3_	1.67	960–962 cm^−1^

Time-resolved spectroscopic measurements can provide useful information on the nucleation and growth processes of minerals. Raman spectroscopy (RS), a form of vibrational spectroscopy, is based on the excitation of molecular vibration by electromagnetic waves. In geosciences, RS is most commonly used as a fingerprinting tool for identifying and characterizing minerals. As such, time-resolved *in-situ* RS presents an easy, rapid and reliable tool with a wide range of applications, e.g., identifying aqueous species, monitoring pathways of mineral formation [24,25], quantitative determination of mineral contents as well as studying the exchange kinetics of e.g., O-isotopes [[26], [27], [28], [29]]. Further, RS is very sensitive to short-range ordered (amorphous) phases. It thus constitutes an excellent tool to study the transformation of amorphous to crystalline materials (e.g. [30]: and references therein). An additional advantage of *in-situ* RS is that it is non-invasive and is thus especially suited for experimental studies with critical sample size or material. No material is consumed during RS analysis, reducing costs and offering a valuable screening tool for quick and reliable analysis.

In this study we investigate the application of high temporal resolution *in-situ* RS as a new and exciting tool for (near) continuous monitoring of mineral (trans-) formation. As an exemplary study we performed calcium phosphate precipitation experiments (T = 20.00 ± 0.01 °C; pH 9.2 ± 0.1) in order to monitor the transformation process of ACP to crystalline HAP. Homogeneous aliquots of the experimental slurry were collected at certain reaction times to follow the chemical evolution of the solution and precipitate and to assess the combined data sets for deciphering transformation kinetics. The precipitates were further investigated for their internal structure to confirm the presence of the respective mineralogical phases.

## Methods

### Experimental setup

**Fig. 1:**
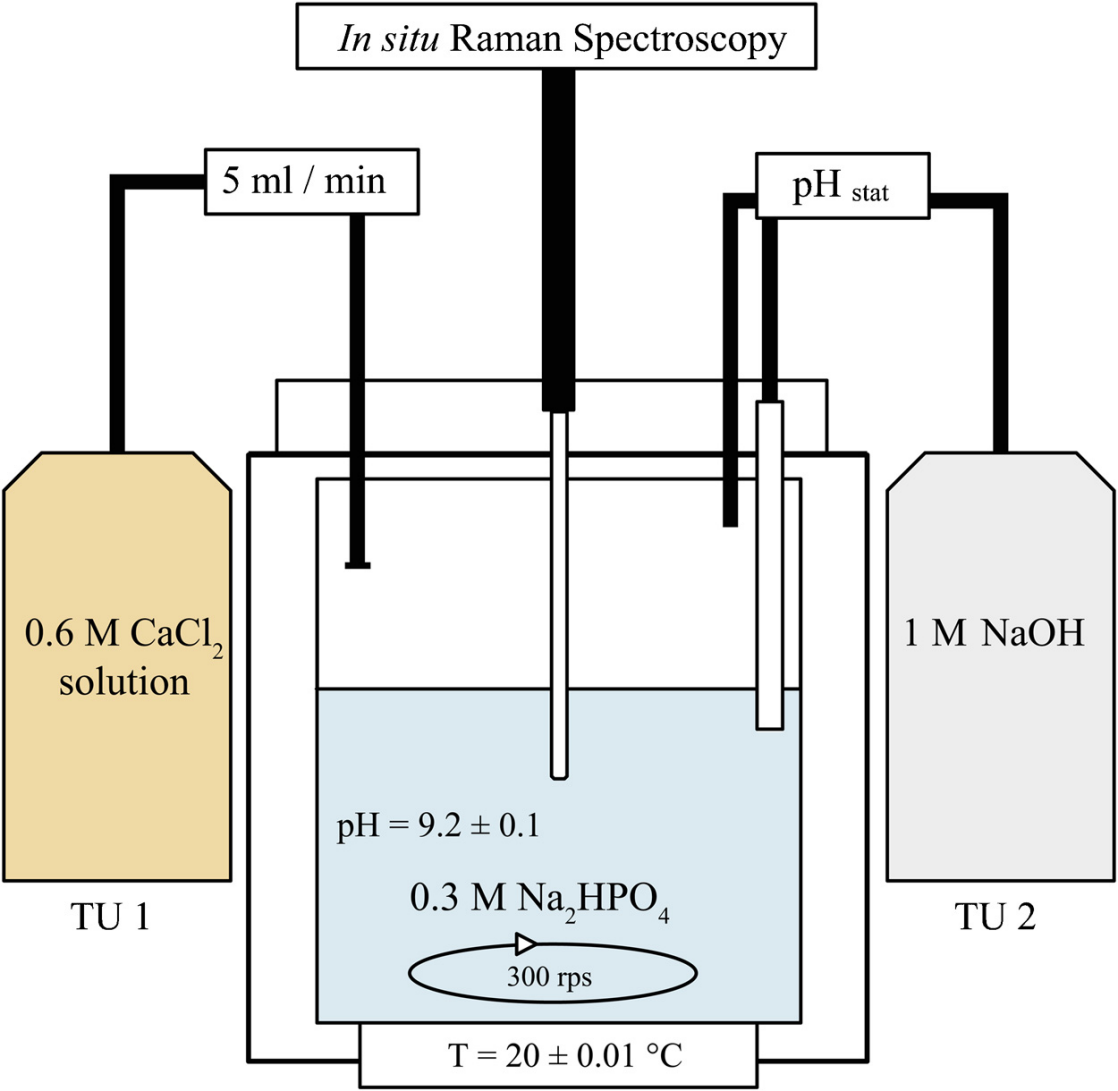
Experimental set up of the calcium phosphate precipitation experiment. Figure adapted from [27]. TU = titration unit.

Calcium phosphate precipitation experiments were performed at constant temperature of 20.00 ± 0.01 °C using an Easy Max^™^ 102 system (Mettler Toledo) equipped with a 150 ml glass reactor and coupled with two titration units (TU), a 702 SM Titrino titrator (Methrom, TU 1) and a TitroLine alpha plus (Schott, TU 2; Fig. 1).

Calcium phosphate precipitation was induced by titrating 50 ml of a 0.6 mol l^−1^ CaCl_2_ solution (TU 1) at a rate of 5 ml min^−1^ to an equal volume of a 0.3 mol l^−1^ Na_2_HPO_4_ solution (glass reactor). The pH was kept constant at pH 9.2 ± 0.1 by titration of a 1 mol l^−1^ NaOH solution (TU 2). Homogenization of the reactive solution was achieved by stirring with a magnetic stirrer at 300 rpm. After 16 h of reaction time the reactive solution was transferred to a gas-tight beaker (Nalgene). Solution was stirred and kept in a temperature-controlled room at 20 °C. During the experiment homogeneous aliquot samples were taken from the reactor and instantly filtered using 0.2 μm cellulose acetate filter. Subsequently, the separated solid samples were lyophilized using a Virtis Benchtop 3 l freeze-dryer (−58 °C; 10 mbar partial vacuum). Sampling times are reported in Table 2.

**Table 2:** Sample name and time of sampling (in min or h) after beginning of CaCl_2_ titration, as well as solution chemistry (pH; Ca, P in mmol l^−1^) and solid chemistry (Ca, P in mmol kg^−1^; Ca/P). Note outlier sample (11) with unusual solid chemistry values; compare Fig. 2.

Sample Name	Sampling time	pH	Solution Ca	[mmol l^−1^] P	Solid Ca	[mmol kg^−1^] P	Ca/P
1	10 min	9.42	174	0.55	8427	6401	1.32
2	17 min	9.34	172	0.37	7851	5796	1.35
3	23 min	9.30	176	0.40	7909	5806	1.36
4	30 min	9.26	165	0.37	8159	5953	1.37
5	35 min	9.23	202	0.33	8028	5871	1.37
6	43 min	9.21	195	0.24	7772	5723	1.36
7	120 min	9.03	167	0.48	7948	5750	1.38
8	180 min	9.21	187	0.25	8145	6014	1.35
9	5 h	9.55	170	0.36	7804	5630	1.39
10	6.7 h	9.26	160	0.19	7235	4955	1.46
11	18 h	9.14	164	0.24	5353	5242	1.02
12	24 h	9.00	180	0.33	8445	6022	1.40

### Analytical procedures

Elemental concentrations of the solid samples (digested in 0.45 mol l^−1^ HNO_3_, p.a.) and solutions were determined by inductively coupled plasma optical emission spectroscopy (ICP-OES) using a PerkinElmer Optima 8300 with an analytical uncertainty of ±2%. Qualitative X-ray diffraction (XRD) analysis for mineralogical characterization of the precipitates were performed using a PANalytical X'Pert PRO diffractometer, equipped with a Co-tube (40 kV and 40 mA), a spinner stage, 0.5° divergence and anti-scattering slits, and a Scientific X'Celerator detector. Randomly oriented preparations were recorded over the range of 4–85°2θ with a step size of 0.008°2 θ.

### Raman instrumentation

*In-situ* Raman spectroscopic analyses were performed using a RAMAN Rxn2^™^ analyser from Kaiser Optical Systems with a Kaiser MR Probe head equipped with a quarter-inch immersion optic. Configuration of the experimental settings was determined using the experiment wizard of the iC Raman^™^ 4.1 software. The wizard automatically guides the user through all configuration steps, including experiment duration, collection of reference spectra and focusing of the instrument. Focusing of the instrument was optimized to a pixel fill of ca. 60%. The pixel fill is the measure of intensity in absolute counts at any given wavenumber with 100% saturation resulting in ca. 65 pixel fill. This was optimized for wavenumber 950– 961 cm^−1^ to an exposure time of 30 s. The Raman spectra were collected in 35 s intervals, consisting of 30 s exposure time and 5 s overtime, for a total of 16 h. After 16 h, Raman spectra were only collected during sampling for shorter intervals, using with the same instrument settings. Spectra were collected in the 100–1890 cm^−1^ region with a resolution of 1 cm^−1^ using a laser beam with an excitation wavelength of 785 nm and a laser power of 400 mW. For comparison, a HAP certified reference material (CRM, Sigma Aldrich) was dissolved in MiliQ. Individual reference spectra of this HAP CRM and of all titrating solutions were collected at 20.00 °C using the same instrumental settings. The obtained reference spectra were only collected for comparison purpose as no automatic calculations, e.g., phase quantifications, were conducted during or after the experiment.

### Data handling

For Raman spectra, first hand data treatment was performed using the iC Raman^™^ 4.1 software (Mettler Toledo), which included baseline correction using the Pearson's method and spectra smoothing using the Savitsky-Golay filter. Intensity was normalized to the intensity of the *ν*4 Raman peak of the reference spectrum. Further data treatment (peak identification and peak fitting) of the collected Raman spectra was performed by the Fityk 0.9.8 (© [31]) non-linear curve fitting and data analysis software. The peaks were identified and fitted employing a Pseudo Voigt function. The Pseudo Voigt function is a convolution of both Lorentzian and Gaussian functions and is often better suited for peak fitting of spectroscopic data. The shape of this fitted Pseudo Voigt function can be quantitatively described by its full width at half maximum (FWHM, compare Fig. 4). The 3D surface plot from Fig. 3 was created using the Origin 9.0.0 (© 1991–2012 OriginLab Corporation) software. For XRD data handling, the baseline was determined manually using the X'Pert HighScore Plus 3.0d (© 2011 PANalytical B.V.) software and subtracted after data collection.

## Results and discussion

### Chemical evolution of experimental solution

**Fig. 2:**
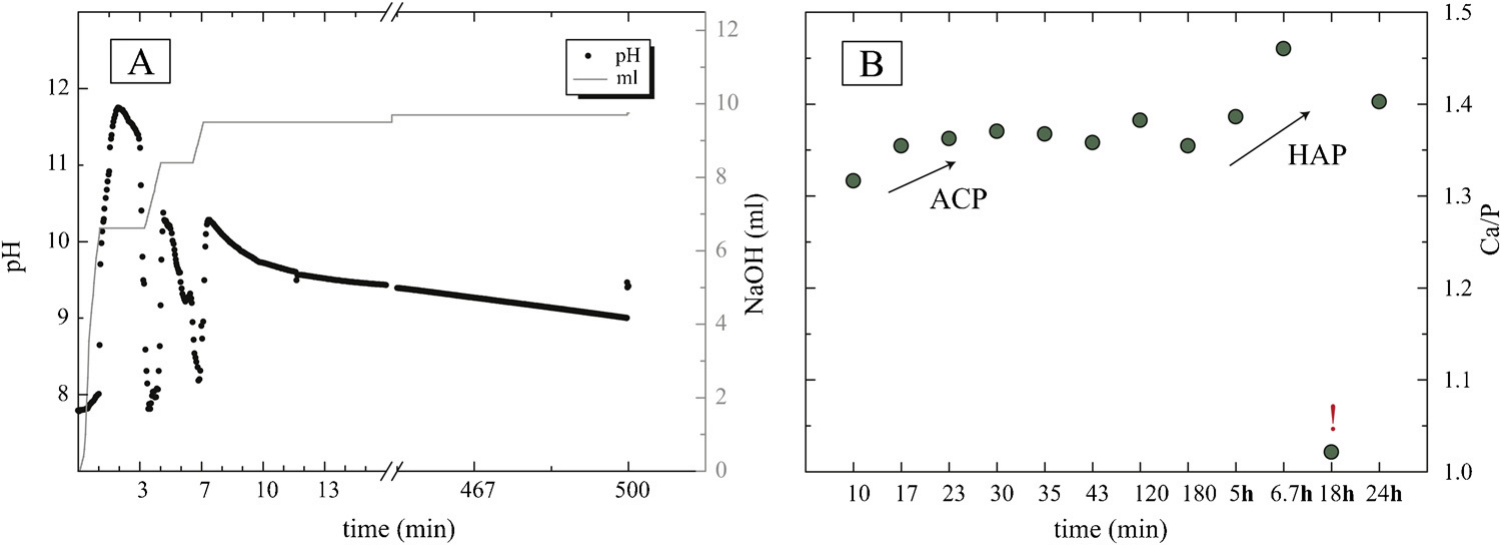
A: pH evolution of the reactive solution and titrated NaOH (ml) of the first 8 h of the experiment. B: Molar Ca/P ratios of solid samples. Note the outlier sample (!) after 18 h, highlighting the consequences of sampling biases.

Elemental compositions of solid and solution samples and pH are reported in Table 2. The addition of the CaCl_2_ solution into the NaHPO_4_ solution induced instant precipitation of calcium phosphate. This initial precipitation and the simultaneous titration of the NaOH solution caused large variations in the pH value within the initial stage of the experiment (Fig. 2). After the addition of the CaCl_2_ solution was stopped (10 min), the pH reached a more constant value, slowly dropping from pH 9.5 towards pH 9 and remained quasi constant at 9.2 ± 0.1 during 24 h of reaction time. Analysis of the reactive solution showed that all PO_4_ is almost quantitatively consumed and only trace amounts of ca. 0.3 mmol l^−1^ were present Table 2. Within this time frame the Ca concentration of the solid remained near constant at 7975 ± 320 mmol kg^−1^. The molar Ca/P of the solid samples ranges from about 1.32 to 1.46, showing a clear increase with time (Fig. 2B). The initial molar Ca/P of ca. 1.3 might indicate the presence of stoichiometric OCP, a transient phase of HAP in alkaline environments [23], or Ca-deficient ACP [32]. In such kind of precipitation experiments non-stoichiometric HAP typically forms at a molar Ca/P of ca. 1.45 ± 0.05 [23]. One solid sample at 18 h has an unusual Ca/P of 1.02 (Fig. 2B), whereas the values of the respective solution are in line with the other results (see discussion in section 3.2).

**Fig. 3:**
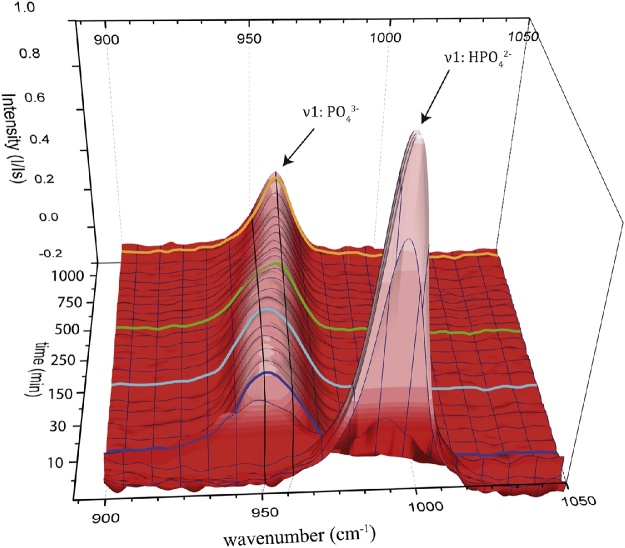
The first sharp peak is produced by the *ν*1 symmetric stretching of the aqueous HPO_4_^2−^ molecule of the Na_2_HPO_4_ solution. Titration of CaCl_2_ immediately induces precipitation of ACP (dark blue line, Fig. 4A), completely consuming all aqueous HPO_4_^2−^. Transformation of ACP into OCP begins after ca. 150 min (bright blue line, Fig. 4B) and to HAP after ca. 500 min (ca. 8 h; green line, Fig. 4C) with complete transition achieved after ca. 1000 min (ca. 18 h; orange line, Fig. 4F–G) indicated by a clear peak sharpening.

**Fig. 4:**
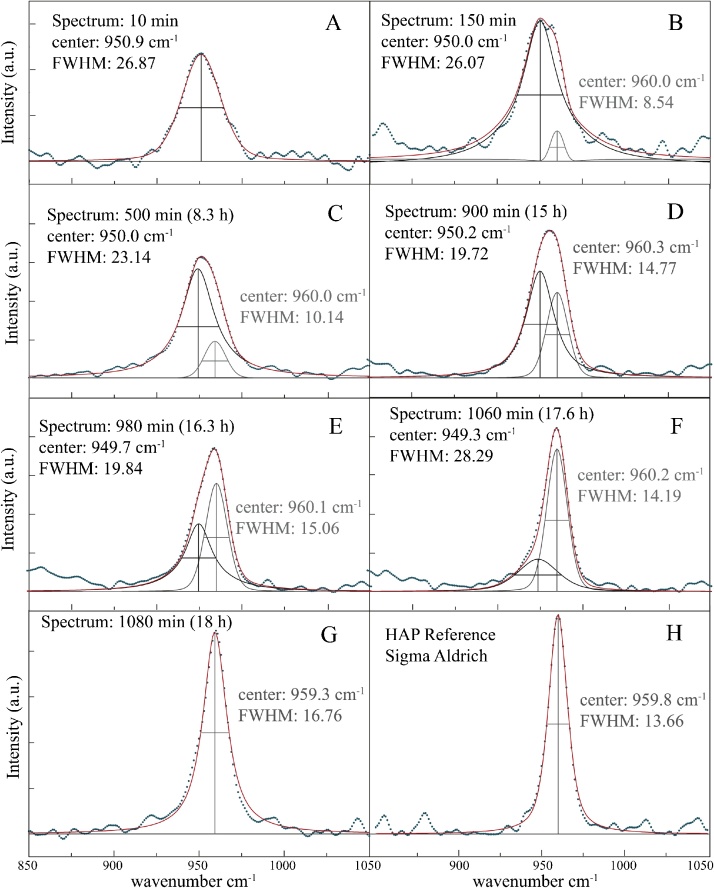
The insets (A–G) show the transformation process of ACP to HAP. The peak fit of selected spectra shows that the observed signal (blue dots) between 150 and 1060 min (Inset B – F) is a convolution (red line) of two interfering peaks at 950 (ACP, black line) and 960 cm^−1^ (HAP, grey line). The transformation process is generally accompanied by a clear peak sharpening recorded in a decreasing FWHM (Inset A–G). The Reference spectrum of well-crystallized HAP (Sigma Aldrich) shows a well-defined sharp peak highlighted by a lowe FWHM (Inlet H).

### *In-situ* Raman spectroscopy and solid characterization

The characteristic vibration bands of PO_4_ groups in HAP crystals are (i) *ν*2 bending of P—O—P at 472 cm^−1^, (ii) *ν*4 bending of P—O—P at 563 and 602 cm^−1^, (iii) *ν*1 stretching of P—O at 960–962 cm^−1^, and (iv) *ν*3 stretching of P—O stretching at 1035–1045 cm^−1^ [33]. For ACP, the most characteristic shift is documented by a 10 cm^−1^ shift of the *ν*1 stretching towards ∼950 cm^−1^ [33]. Thus, the transformation of ACP to HAP is characterized by a shift of the *ν*1 symmetrical band from a broad peak at ca. 950 cm^−1^ towards a narrow peak at 960 cm^−1^ [7]. The observed peak is thus a convolution of the individual peaks at 950 cm^-1^ (black line in Fig. 4) and 960 cm^−1^ (grey line in Fig. 4). Employing the peak fitting method using a Pseudo Voigt fit for the peak between 850 cm^−1^ and 1050 cm^−1^ these individual peaks can be separated (Fig. 4). In the initial solution, the *in-situ* Raman spectra showed a distinct peak of the *ν*1 band of the (HPO_4_^2−^)_aq_ molecule at 990 cm^−1^ (Fig. 3). After the onset of CaCl_2_ titration, the intensity of this band quickly decreased within the first 3–5 min and a peak at 950 cm^−1^ appeared, indicating the formation of ACP (compare dark blue line in Fig. 3, Fig. 4, Inset A). In a later stage, after about 150 min the peak begins to shift towards the 960 cm^−1^ band, indicating the presence of crystalline HAP (compare bright blue line in Fig. 3, Fig. 4, Inset B). Between 500 min (ca. 8 h, compare green line in Fig. 3) and 900 min (15 h) the magnitude of the 960 cm^−1^ band of HAP increases shifting the center of the peak further towards 960 cm^−1^ (Fig. 4 Inset C–D). After ca. 980 min (16.3 h) the intensity of the 960 cm^−1^ HAP band is stronger than of the 950 cm^−1^ ACP band (Fig. 4 Inset E), with a shoulder remaining until 1060 (17.6 h, Fig. 4 Inset F). Shortly after, this shoulder is not present or below detection limit, indicating that the majority of the solid phase consists of HAP but traces of ACP cannot be excluded (compare orange line in Fig. 3, Fig. 4 Inset G), similar to that of the certified reference material (Sigma Aldrich, Fig. 4, Inset H). Transformation of ACP into HAP was accompanied by a clear peak sharpening. This sharpening was recorded by a decreased FWHM of the Pseudo Voigt function from 26.87 cm^−1^ of the amorphous phase after 10 min (Fig. 4, inlet A) to 16.76 cm^−1^ of the crystalline state (Fig. 4, Inset G).

Chemical composition of the solid samples suggests that the transient phase during the initial stages of the experiment up to 5 h (300 min) is stoichiometric OCP. However, the *ν*1 P—O stretching of OCP is at ca. 955 cm^−1^ [34]. The peak shift of the *ν*1 band to 955 cm^−1^ is only observed after 5 h and as demonstrated in Fig. 4 caused by interfering peaks of ACP and HAP at 950 and 960 cm^−1^ respectively. Although OCP is often observed as an intermediate phase in the formation of HAP [35,36], this could not be confirmed in the present study. However, OCP is usually observed in experiments at pH < 7 [37,38]. Therefore, the absence of OCP could be due to the considerably high pH in this study and may be related to the prevailing aquo-species, which within this experiment is HPO_4_^2-^, whereas at pH < 7 H_2_PO_4_- is the dominant species. In similar terms the formation of amorphous calcium carbonate system mainly occurs at elevated pH where aqueous CO_3_^2−^ prevails compared to lower pH where HCO_3_- is the dominant aqueous species [39]. Additionally, it is worth noting here that temperature may also have an effect on the crystallization pathways of ACP. As it has been reported earlier by Combes & Rey [7] and Eanes [8] at formation temperatures exceeding 37 °C stability of ACP drastically decreases to <30 min. This temperature effect on the formation via amorphous precursors is also met in the CaCO_3_ system, where ACC can be a precursor at temperatures below 25 °C [39], whereas this is not the case when mineral forms above 40 °C [40].

**Fig. 5:**
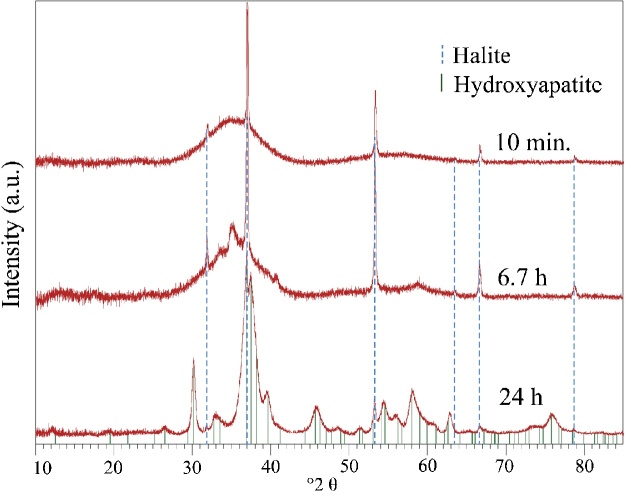
Time-resolved, stacked XRD patterns confirm the amorphous state after 10 min and record the progressive crystallization as a function of time, indicated by development of distinct peaks. Halite (NaCl) co-precipitates as an artifact of the chosen method.

Time-resolved XRD patterns of the collected solids are displayed in Fig. 5. Those confirm the presence of ACP in the reactive solution shortly after CaCl_2_ titration (10 min). Comparison of the time integrated samples clearly shows the increasing crystallinity and thus the degree of ordering in the solid samples. After 10 min, the XRD pattern shows a very broad peak at ca. 36°2θ, while after 405 min (6.7 h) crystalline material, enough for detection, can be observed. After 24 h, the XRD pattern indicates distinct peaks, coherent with crystalline HAP. Although the outlier sample at 18 h does record unusual Ca/P in the solid sample, the XRD patterns (not shown here) and Raman spectra (Fig. 4F, G) do not record any unusual patterns. Crystallization of ACP to HAP is accompanied by an increase of Ca. However, this outlier sample records unusually low Ca values. This and the fact that XRD patterns of the same sample was normal, renders premature crystallization due to e.g. delayed lyophilization unlikely. The unusual Ca value is thus likely attributed to contamination during sample dilution.

## Summary and concluding remarks

Previous experimental studies on HAP formation suggested that in aqueous alkaline media, OCP occurs as an intermediate phase during the transformation of ACP to HAP [41]. In this context, it has been shown that OCP precipitates rapidly by hydrolysis of ACP, forming a layered structure comprising an apatite and hydroxide layer. In an autocatalytic reaction, OCP then slowly transforms to HAP [41]. In the present study, *in-situ* RS was successfully applied to detect the transformation of ACP to HAP at a high time-resolution of Raman spectra of 35 s. Although indicated by chemical data of the solid samples, the presence of OCP as an intermediate phase could not be confirmed. The presence of the respective peak of the v1 P—O band at ca. 955 cm^−1^ is likely produced by two overlapping peaks of ACP (950 cm^−1^) and HAP (960 cm^−1^), highlighting the necessity of RS. The transformation from amorphous (ACP) to nano-material towards crystalline material (HAP) is accompanied by (1) an increased degree of ordering (Fig. 5), along with (2) an increase of the molar Ca/P ratio of the precipitating solids (Fig. 2) and (3) a clear peak sharpening of the *ν*1 Raman band (Fig. 4). *In-situ* Raman spectroscopy facilitates the visualization of this increase in crystallinity and ordering, i.e., lattice ordering, in nearly real time. *In-situ* Raman spectroscopy thus facilitates the (near) continuous monitoring of experimental solutions at high temporal resolution and has a clear advantage over manual sampling. Individually extracted samples can thus only offer ‘snap-shots’ of a dynamic temporal evolution. The outlier sample after 18 h further highlights the sensitivity of sample results due to small sampling biases (Fig. 2). This is especially important when working with meta-stable materials, underlining the necessity of *in-situ* Raman spectroscopy. The given protocol can be used in both, industrial applications as e.g., a quality screening tool, or for research applications such as *in-situ* reaction monitoring in aqueous media to follow and assess transformation processes, phase identification or crystallinity. In future studies, this protocol could also be combined with computed peak fitting, similar as shown in Fig. 4, to quantify species abundances and calculate conversion kinetics.
